# Dataset of annotated food crops and weed images for robotic computer vision control

**DOI:** 10.1016/j.dib.2020.105833

**Published:** 2020-06-11

**Authors:** Kaspars Sudars, Janis Jasko, Ivars Namatevs, Liva Ozola, Niks Badaukis

**Affiliations:** a Institute of Electronics and Computer Science, Dzērbenes str.14, Riga LV-1006, Latvia; b Institute for Plant Protection Research `Agrihorts’, Latvia University of Life Sciences and Technologies, P. Lejiņa str. 2, LV-3004 Jelgava, Latvia

**Keywords:** Computer vision, Object detection, Image annotation, Precision agriculture, Crop growth and development

## Abstract

Weed management technologies that can identify weeds and distinguish them from crops are in need of artificial intelligence solutions based on a computer vision approach, to enable the development of precisely targeted and autonomous robotic weed management systems. A prerequisite of such systems is to create robust and reliable object detection that can unambiguously distinguish weed from food crops. One of the essential steps towards precision agriculture is using annotated images to train convolutional neural networks to distinguish weed from food crops, which can be later followed using mechanical weed removal or selected spraying of herbicides. In this data paper, we propose an open-access dataset with manually annotated images for weed detection. The dataset is composed of 1118 images in which 6 food crops and 8 weed species are identified, altogether 7853 annotations were made in total. Three RGB digital cameras were used for image capturing: Intel RealSense D435, Canon EOS 800D, and Sony W800. The images were taken on food crops and weeds grown in controlled environment and field conditions at different growth stages

Specifications Table

**Table:** 

**Subject**	Agronomy and Crop Science, Computer Vision and Pattern Recognition.
**Specific subject area**	Object classification, Object detection, Object recognition, Crop growth and development.
**Type of data**	Image. Annotations.
**How data were acquired**	The data was acquired by capturing images with a resolution of 720 × 1280 × 3, 1000 × 750 × 3, 640 × 480 × 3, 640 × 360 × 3 and 480 × 384 × 3 pixels in a controlled and unregulated environment using the Canon EOS 800D, and Sony W800 digital cameras and the Intel RealSense D435 camera. Images were manually annotated by using software importing: python os, cv2, sys, xml.etree.ElementTree.
**Data format**	Raw images:.jpg format, manually annotated images: .xml files
**Parameters for data collection**	Data was acquired by capturing images in field conditions and in a controlled environment.
**Description of data collection**	Dataset consists of two directories. Directory *images*) 1118 food crops and weed images and directory *annotations,* i.e. their 1118 counterpart annotation XML files, which can be included 7853 annotations of two classes: food crops (six species), 441 annotations and weed (eight species), 7442 annotations.
**Data source location**	Municipalities: • Jelgava (controlled environment) • Ķekava parish (open field) • Rūjiena parish (open field) • Krimulda parish (open field) • Country: Latvia
**Data accessibility**	With an article

**Value of the Data**

• The dataset presents images of food crops and weed in their seedling growth stages and, respectively, their manually annotated images. It can be useful for agronomists and researchers in different fields for precision agriculture and computer vision tasks.
• The dataset is open access and can be used for future researchers and engineers for constructing their own food crops and weed recognition algorithms.
• The dataset can be used for benchmarking deep learning algorithms, recognizing objects, constructing models and navigate robotics.
• The dataset can furthermore be used to train, tests and validate convolutional neural networks.

## 1 Data description

The data presented in this paper provides images of food crops and weed in their early growth stage as seedlings. Recently, machine learning, deep learning, and image-processing have shown great potential in progressing the digital capabilities need for the future of agriculture [1]. The robots and the vision machines need to be able to precisely and detect a wed from the useful plants [2]. To establish a plausible weed/crop detector in robotic weed control management, it must be focused on detection, mapping, guidance, and control technologies based on using digital images. The data presented in the dataset is targeted to identify food crops and weed in an image. The dataset consists of **1118** images (.jpg files) along with **7853** XML manually annotated annotations (.xml files) in them. The type of images is digital, and the format is a bitmap (raster) color three samples per point, RGB. Canon EOS 800D and Sony W800 digital cameras and Intel RealSense D435 cameras were used to take photos. Images in dataset have following dimensions (physical resolution, dimensions): 720 × 1280 × 3, 1000 × 750 × 3, 640 × 480 × 3, 640 × 360 × 3 and 480 × 384 × 3. The dataset is divided into two main classes: food crops 411 annotations and weeds 7442 annotations. An Intel RealSense D435 camera and Sony W800 digital camera were used to acquire images in field conditions, but Canon EOS 800D digital camera was used in a controlled environment. The data is organized into two directories: images and annotations.

## 2 Experimental design, materials, and methods

### 2.1 Literature search

Generally, the scientific community struggles with the shortage of “open” artificial systems and datasets that can be accessed by all stakeholders and researchers to address their tasks. Machine learning makes a good promise in plant identification issues, and datasets play a crucial role in today`s computer vision research tasks [3]. These technologies are used in different fields of agriculture: crop disease detection, weed classification and identification, plant seedling classification fruit identification and accounting, water resources and soil management, weather forecasting (climate), etc. [4], [5], [6], [7], [8]. Perhaps the greatest obstacle for widespread uptake of robotic weed control is the robust classification and detection of weed species in their natural environment [9]. Most of the datasets are only used for classification tasks, and there is no annotation for the detection tasks [10]. In order to effectively train networks of neurons, a single regional food crops and weed dataset must be developed, including ground truth bounding boxes. The more information will be included in these datasets, the more efficient artificial intelligence systems will work for robotic weed control management, more precise will be plant growth and more effective will be allocation of limited resources.

**Fig. 1:**
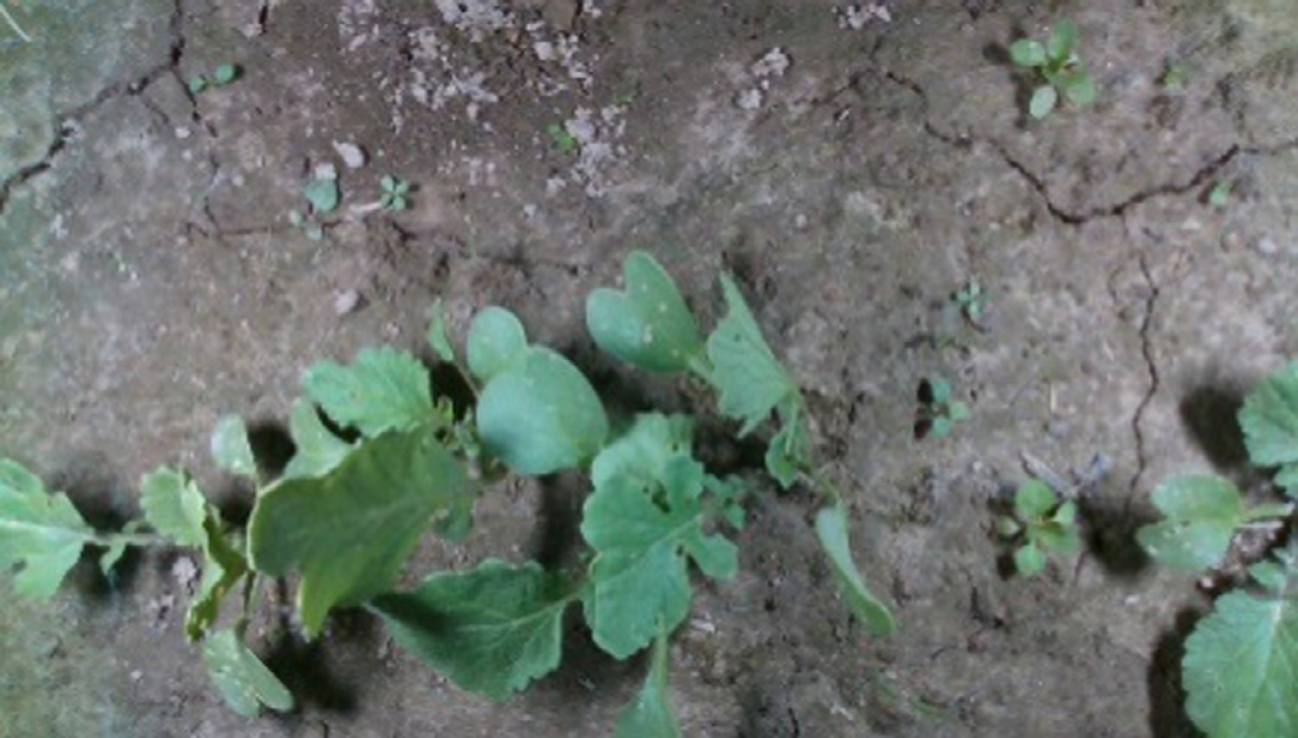
Raw images of food crops and weed of the dataset.

**Fig. 2:**
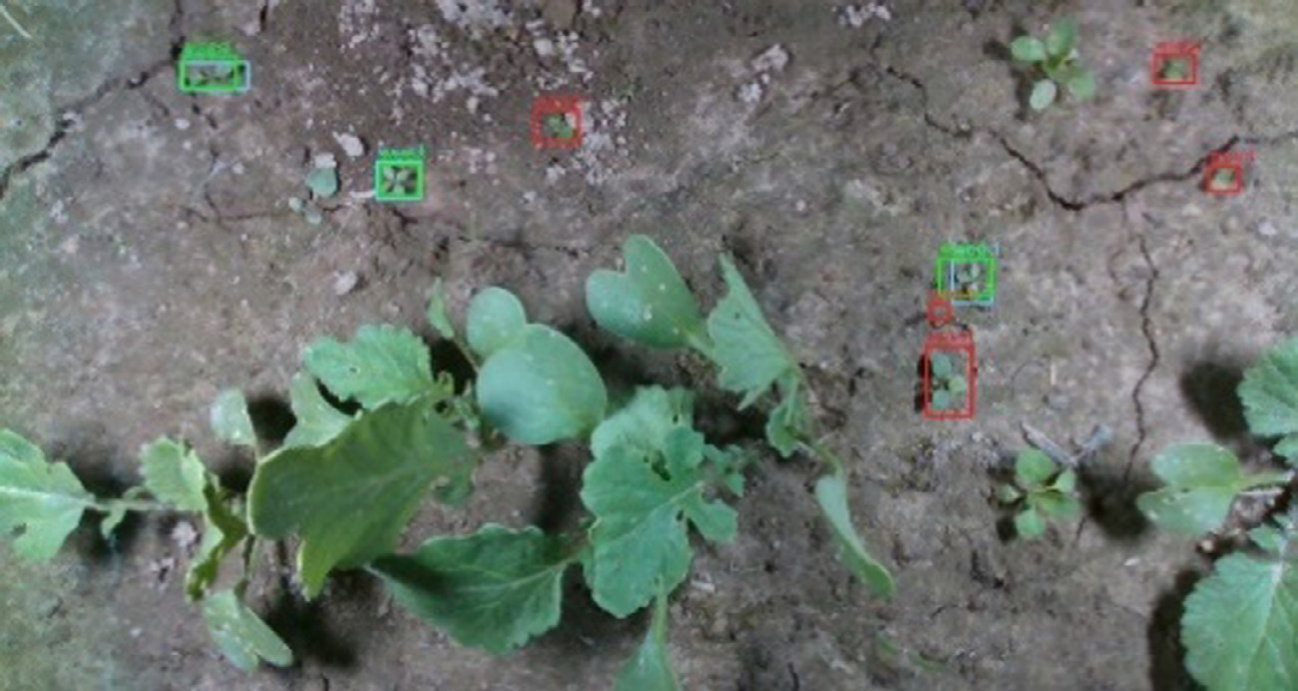
Annotated images of the crop and weed with the colored bounding boxes.

Table 1 shows how the dataset is distributed among food crops and weed species. 14 essential food crops and weed species that occur in arable fields of Latvia (the Baltic region in the Northern Europe) have been selected for dataset. The abbreviation in the table refers to the species ID, which element-wise refers to the designation of taxonomy classification, family, and common name of the class of food crops and weeds. Fig. 1 displays the raw (field) image samples of dataset. All the dataset`s raw images are in .jpg. format. The raw images are given in original RGB. Digital cameras were used in two environments to capture the images – in a greenhouse and in field conditions. The cameras were pointed vertically towards the ground when the image was being taken. To ensure the fix camera height from the ground, the cameras was mounted on top of a three-point photostat in a greenhouse and platform mounted in filed conditions. Images of food crops and weeds were taken once a day during their early growth stage starting the first stage of the species growth. Fig. 2 displays example of images extracted from the raw image set, along with ground truth for food crop or weeds. For every raw image of the dataset the annotation images are given. Human experts annotated all raw images manually. All raw images were annotated by human experts. The experts were asked to mark food crop and weed species with closed polygons and to assign a type (food crop or weed) to each polygon. All pixels that lie inside a polygon inherit the label from the polygon. The annotated dataset contains both the polygon information and the crop /weed annotation images.

**Table 1:** List of food crops and weed species available in the dataset.

Abbreviation	Taxonomic classification	Family	Common name	Class
CA	*Chenopodium album* L.	*Amaranthaceae*	Goosefoot	*Weed*
GA	*Galium aparine*	*Rubiaceae*	Catchweed	*Weed*
TA	*Thlaspi arvense*	*Brassicaceae*	Field pennycress	*Weed*
CB	*Capsella bursa-pastoris*	*Brassicaceae*	Shepherd`s pursue	*Weed*
MI	*Matricaria perforata*	*Asteraceae*	Field chamomile	Weed
PC	*Polygonum convolvulus*	*Polygonaceae*	Wild buckwheat	Weed
VA	V*iola arvensis*	*Violaceae*	Field pansy	Weed
GP	*Galinsoga parviflora*	*Asteraceae*	Quickweed	Weed
BV	*Beta vulgaris*	*Amaranthaceae*	Common beet	*Crop*
DC	*Daucus carota* var. *sativus*	*Apiaceae*	Carrot	*Crop*
CPS	*Cucurbita pepo* subsp. *pepo*	*Cucurbitaceae*	Zucchini	*Crop*
CP	*Cucurbita pepo*	*Cucurbitaceae*	Pumpkin	*Crop*
RSS	*Raphanus sativus* var. *sativus*	*Brassicaceae*	Radish	*Crop*
RSN	*Raphanus sativus* var. *niger*	*Brassicaceae*	Black radish	Crop

### 2.2 Experimental set up

The species of the food crops were chosen based on their popularity among consumers in Latvia and the necessity to implement intensive weed management solutions.

Two types of images are included in data set: (i) images of food crops and weeds that have been cultivated in vegetation pots in controlled greenhouse conditions; (ii) images of food crops and weeds from open field conditions. Images of plants from greenhouse were taken at the Scientific Institute for Plant Protection Research “Agrihorts”, University of Life Sciences and Technologies of Latvia, Jelgava, Latvia. Images from field conditions are from three locations in Latvia: Kekava, Rujiena, and Krimulda. The digital images were captured with perspective projection over plants.

To build the dataset, common weed species found in vegetable fields were selected, 8 weeds: goosefoot (*Chenopodium album*), catchweed (*Galium aparine*), field pennycress (*Thlaspi arvense*), shepherd's purse (*Capsella bursa-pastoris*), field chamomile (*Matricaria perforata*), wild buckwheat (*Polygonum convolvulus*), field pansy (*Viola arvensis*), quickweed (*Galinsoga parviflora*). There were 6 food crops selected: beetroot (*Beta vulgaris*), carrot (*Daucus carota* var. *sativus*), zucchini (*Cucurbita pepo* subsp. *pepo*), pumpkin (*Cucurbita pepo*), radish (*Raphanus sativus* var. *sativus*), black radish (*Raphanus sativus* var. *niger*). The list of food crops and weeds is presented in Table below:

In a greenhouse, plants were grown in vegetation pots under natural and artificial light. The peat substrate was used for soil preparation with such characteristics: pH 6.0, moisture content <65%, peat fraction <20.0 mm, N 12.0%, P2O5 14.0%, K2O 24.0%, Te 1.0 kg m-3. In each vegetation pot, the seeds of the plants were sown in one to two rows at a distance between them of 2.0 – 5.0 cm. The seedling boxes were watered once to twice per week. Temperature was set to +20.0 Co daytime (8:00 a.m. – 8:00 p.m.), and +15.0 Co during the night (8:00 p.m. – 8:00 a.m.), humidity <50%. Additional to natural light, LED lamps (Philips Lightning IBRS, 180 W) were used for plant cultivation with illumination period from 6:00 a.m. – 8:00 p.m.

Photos of vegetation boxes were taken with additional light and a three-point supporting photostat. A distance of 30 cm was set from the lens of the camera to the surface of the vegetation box. Once a day, images of plants were taken starting from the first stage of the plant development.

Images of plants in field conditions were taken in the organic vegetable farms in three locations specified above before weeding activities carried out by farmers. To capture images a platform mounted on four wheels was constructed. A digital camera was attached in the middle of the platform with the lens directed downwards. The platform was moved across the field in different directions to take photos of the plants. Afterwards, weeds and crops at early growth stages were manually marked in the pictures with ground truth bounding boxes, see Fig. 2.

Python-based software was created to make annotations of the images. Each image labeled individual food crops and weed, taking into consideration that the area of the green surface of the crop leaves doesn't overlap with the leaves of other crops. The labels of each image, with their coordinates, were stored in the document folder *annotations*. Annotated XML files are compatible with annotation rules used in popular Pascal VOC dataset: http://host.robots.ox.ac.uk/pascal/VOC/voc2007/

## Declaration of Competing Interest

The authors declare that they have no known competing financial interests or personal relationships that could have appeared to influence the work reported in this paper.

## References

[1] Khaki S., Pham H., Han Y., Kuhl A., Kent W., Wang L. (2020). Convolutional neural networks for image-based corn kernel detection and counting. Sensors.

[2] D. Nkemelu, D. Omeiza, and N. Lubalo, Deep convolutional neural network for plant seedlings classification, cs.CV, 2018. URL arXiv:11811.08404v1

[3] Yan J., Zhang X., Lei Z., Liao S., Li S.Z. (June 2013). Robust multiresolution pedestrian detection in traffic scenes. Proceedings of the IEEE CVPR.

[4] Boulent J., Foucer S., Thean J., St-Charles P.L. (July 2019). Convolutional neural networks for the identification of plant diseases. Front Plant Sci..

[5] Jeon W.S., Rhee S.Y. (March 2017). Plant leaf recognition using a convolutional neural network. Int. J. Fuzzy Logic Intell. Syst..

[6] D. Nkemelu, D. Omeiza, and N. Lubalo, Deep convolutional network for plant seedlings classification, arXiv: 1811.08404v1 [cs.CV]20 Nov 2018

[7] Koirala A., Walsh K.B., Wang Z., McCarthy C. (Februry 2019). Deep learning for real-time fruit detection and orchard fruit load estimation: benchmarking of `MangoYOLO`. Precis. Agric..

[8] Liakos K.G., Busato P., Moshou D., Pearson S. (2018). Machine learning in agriculture: a review. Sensors.

[9] Steward B.L., Gai J., Tang L., Billingsley J. (2019). The use of agricultural robots in weed management and control. In Robotics and Automation for Improving Agriculture.

[10] Louargant M., Jones G., Paoli J.N., Maillot T., Gée C., Villette S. (2018). Unsupervised classification algorithm for early weed detection in row-crops by combining spatial and spectral information. Remote Sens..

